# Homologous Recombination in Negative Sense RNA Viruses

**DOI:** 10.3390/v3081358

**Published:** 2011-08-04

**Authors:** Guan-Zhu Han, Michael Worobey

**Affiliations:** Department of Ecology and Evolutionary Biology, University of Arizona, Tucson, AZ 85721, USA; E-Mail: worobey@email.arizona.edu

**Keywords:** homologous recombination, negative sense RNA viruses, evolution

## Abstract

Recombination is an important process that influences biological evolution at many different levels. More and more homologous recombination events have been reported among negative sense RNA viruses recently. While sporadic authentic examples indicate that homologous recombination does occur, recombination seems to be generally rare or even absent in most negative sense RNA viruses, and most of the homologous recombination events reported in the literature were likely generated artificially due to lab contamination or inappropriate bioinformatics methods. Homologous recombination in negative sense RNA viruses should be reported with caution in the future, and only after stringent quality control efforts. Moreover, co-infection experiments should be performed to confirm whether recombination can occur.

## 1. Introduction

Negative sense RNA viruses (NSVs) differ widely in morphology and host interactions. NSVs include a myriad of important pathogens such as influenza viruses, measles virus (MeV), Newcastle disease virus (NDV), rinderpest virus (RPV) and many others infecting humans, several wild and domesticated animals, and plants. NSVs have varied genome structures and are classified into seven families, *Rhabdoviridae*, *Paramyxoviridae*, *Filoviridae*, *Bornaviridae*, *Orthomyxoviridae*, *Bunyaviridae*, and *Arenaviridae* [1]. The first four families are characterized by non-segmented genomes and constitute the order Mononegavirales [1]. The latter three families have genomes comprising multiple negative sense RNA segments.

RNA viruses show high mutation frequencies partly because of a lack of the proofreading enzymes that assure fidelity of DNA replication [2,3]. While mutation is the ultimate source of genetic variation, recombination can act on mutation to shape the genetic structure of populations. Two distinct mechanisms can generate viral recombinant genomes [4,5]. Reassortment occurs when two or more multipartite viruses co-infect a single host cell and exchange discrete RNA segments to form genetically novel progeny viruses. Antigenic shift in influenza virus, a reassortment event that generates a novel combination of the hemagglutinin (HA) and neuraminidase (NA) antigens, provides an important example for this sort of recombination [6]. Recombination, the other process, can be defined as the intramolecular exchange of genetic information between two nucleotide sequences [7]. In other words, it occurs when one contiguous stretch of RNA is formed as a mosaic from more than one ‘parent’. Recombination events can occur between different genes (non-homologous/intergenic recombination) or between alleles of the same gene (homologous/intragenic recombination), although new terminologies have been proposed [8,9]. In this review, we focus on homologous recombination that involves two similar or closely related RNA molecules with extensive sequence homology [8].

During the past two decades, many recombination analysis and detection methods have been developed. These techniques differ greatly in approach and applicability, but may be tentatively classified into five nonexclusive general categories: Similarity, distance, phylogenetic, compatibility, and nucleotide substitution distribution methods [5]. A comprehensive list of these recombination analysis and detection programs can be found in the following website [10]. These methods generally focus on one or more aspects of recombination, including estimating the recombination rate, identifying parental and recombinant sequences, and mapping recombination breakpoints. Different recombination analysis and detection methods show distinct performance and the relative performance has been evaluated by both theoretical and empirical studies, leading to the suggestion that definitive conclusions about the presence of recombination should not be derived on the basis of a single method [7,11].

## 2. False Positive Recombination Signals

False positive recombination signals can be generated artificially. The most important alternative hypothesis for putative recombination is laboratory contamination. If samples from co-infected hosts contain RNA genomes present from multiple viruses, subsequent PCR amplification, especially with primers from different regions, may result in specific segment sub-regions being amplified from different viruses, then concatenated *in silico*, giving the appearance of recombination where none has occurred [12]. Errors can also occur during data processing. Zhang and Liu recently reported a single recombination event between mumps virus (MuV) wild type Drag94 and the vaccine strain L3/Russia/Vector, leading to a putative recombinant 9218/Zg98 strain, after analyzing 30 complete viral genomes retrieved from GenBank [13]. The sequence of strain 9218/Zg98 is 100% identical to strain L3/Russia/Vector in the recombination region. Both strains were sequenced in the same laboratory. Subsequently, the sequence submitter discovered that there was an error during the submission process [14–16].

Template jumping between different templates during PCR amplification can also result in apparent recombination. Some researchers argue that recombinants only represent a minor portion of the PCR amplicon and the resulting sequence is a consensus sequence of the overall population of amplicons and thus exclude this possibility of recombination [17]. However, previous studies have indicated that, for example, ∼5% of the 16S sequences within curated collections are anomalous or suspect, with chimeras accounting for the majority of problematic sequences [18]. PCR amplification with samples infected with genetically diverse *Plasmodium* parasites generated ample *in vitro* recombinants [19]. This observation indicates that recombinant sequences generated by sequencing PCR products are not rare, and there is no reason to suspect that RNA viruses would be immune to similar pitfalls.

Bioinformatic methods can be another source of artifacts that look deceptively similar to genuine recombination. All recombination methods can infer false positives, albeit often at low levels [7]. For instance, lineage-specific rate variation can result in false positive recombination signals. An important example lies in the 1918 Spanish flu virus. Homologous recombination was proposed to occur in the HA gene of the 1918 Spanish flu virus [20]. However, this apparent recombination event was later shown to be the result of differences in the substitution rate between HA1 and HA2 [21]. Because the HA1 domain evolves so much faster than the HA2 domain in human (but not swine) influenza A viruses, the 1918 strain appears to be closer to classical swine influenza strains than to recent human strains, based on approaches that rely primarily on sequence similarity. In such cases, a phylogenetic approach that can infer shared ancestry, even when some lineages evolve relatively slowly or rapidly, is key. This problem goes beyond RNA viruses and was encountered, for instance, when crude methods based on sequence similarity, rather than phylogenetic relatedness, led to erroneous conclusions of lateral gene transfer from bacteria to vertebrates including humans [22].

## 3. Guidelines for Identifying Recombination

Boni *et al*. recently proposed guidelines for identifying homologous recombination in influenza A virus [12]. Following their lead, here we propose a set of relatively simple guidelines and recommendations for identifying recombination in NSVs, which we believe can also be applied to other kinds of viruses. These guidelines and recommendations are not foolproof and are not meant to replace healthy skepticism of results suggestive of recombination in RNA viruses. The key point is that researchers must explain to themselves, to reviewers, and to others why sequences should be believed to be authentic recombinants rather than ignoring the very real possibility of artifacts of one sort or the other.

### 3.1. Excluding the Possibility of Laboratory Contamination

Apparently mosaic sequences should be reproducible. If possible, sequences should be generated after plaque purification or limiting dilution, and multiple independent extractions followed by amplification and sequencing should be performed. Single-genome amplification (SGA), which has been used extensively to characterize the genetic identity and complexity of human and simian immunodeficiency viruses [23–27], is highly recommended.

Evidence that recombinant sequences form a distinct circulating lineage, with readily identifiable parents, and have been transmitted among multiple individuals in a population, are strong indicators of authentic recombination events. If one or more of the putative parental strains are ones that have commonly been used in the laboratory, or if recombination break points lie near primer loci, checking the authenticity of sequences by repeating extractions and sequencing assays becomes particularly important.

### 3.2. Excluding the Possibility of False Positive Signals Due to Bioinformatics Errors

Definitive conclusions about the presence of recombination should not be derived on the basis of a single method [7]. Phylogenetic analysis provides a robust, informative, and necessary test of the recombination hypothesis. The gold-standard bioinformatics approach for demonstrating the presence of recombination is a set of statistically incongruent phylogenetic trees for different genomic regions [12]. Given the intrinsic uncertainty in solving phylogenetic relationships, statistical procedures, such as the Shimodaira-Hasegawa test [28], are highly recommended for performing topology testing of phylogenies.

Alignment errors or uncertainty can also lead to false positive recombination signals [12], due to either similarity shifts or phylogenetic discrepancies caused by misaligned sequences. Therefore, sequence alignments should be visually analyzed for obvious alignment errors before carrying out recombination analysis [12].

## 4. Recombination Events in Negative Sense RNA Viruses

Frequent generation of defective interfering (DI) RNA, which can be viewed as one form of non-homologous recombination has been observed in NSVs [29]. However, extensive co-infection experiments performed many years ago, with a variety of markers, failed to reveal evidence of homologous recombination in Newcastle disease virus (NDV) and vesicular stomatitis virus (VSV), suggesting that recombination is either very rare or nonexistent in some NSVs [2,30]. Decades later, two studies showed unambiguously that recombination can occur in both non-segmented and segmented NSVs [31,32]. Transfection-mediated homologous recombination *in vitro* resulted in the rescue of functionally competent Tula hantavirus with a recombinant S RNA segment [31]. And mixed infection *in vitro* by human respiratory syncytial virus (RSV) yielded a viable, helper-independent mononegavirus [32]. However, the isolation of only a single recombinant RSV under optimized conditions suggests that recombination is rare indeed in RSV [32]. Systematic phylogenetic analyses have suggested a low rate of recombination in negative sense RNA viruses [33]. However, with the increasing availability of viral sequences, more and more recombination events have been reported in NSVs recently. Table 1 provides what is to our knowledge the complete list of recombination events reported in NSVs to date, which is discussed in turn below. Until now, homologous recombination is reported only in animal-infecting NSVs and, to our knowledge, there is no report on recombination in plant-infecting NSVs.

### 4.1. Influenza Virus

Influenza viruses undergo various forms of non-homologous recombination, albeit rarely [69–71]. However, homologous recombination is hotly debated in influenza viruses. Homologous recombination was proposed to occur in the HA gene of the 1918 Spanish flu virus [20]. This intragenic recombination was speculated to have resulted in the increased virulence associated with the Spanish flu pandemic. As described above, this apparent recombination event was actually an artifact of differences in the substitution rate between the HA1 and HA2 regions [21]. Recently, homologous recombination is suggested to take place between influenza virus strains sharing high sequence similarity [54]. However, the p-values obtained from standard recombination detection algorithms are ambiguous, making the claims unconvincing.

Several recent studies have reported new evidence for recombination in avian, human, and swine influenza A virus lineages [51–54]. For example, a clade of three circulating H9N2 avian influenza viruses with similar mosaic PA genes descended from H9N2 and H5N1 was reported and showed that recombination may have occurred. Further back in time, one experimental study that has been largely overlooked revealed that, following mixed infection of two viral strains, one reassortant contained a nucleoprotein (NP) segment with sequences derived from each parental virus as a result of a recombination event [55].

These findings, however, must be viewed in the context of recent large-scale analyses of influenza A, B, and C viruses that showed that recombination is exceedingly rare. Most recombination reported in the literature [51–54] has involved sequences retrieved from public databases such as GenBank. However, typically in such cases sample handling and amplification and sequencing approaches are of unknown quality and some putative recombinants were isolated decades apart from their parental sequences, which make the recombinant sequences highly suspect, to say the least [12]. An analysis of 8307 publicly available full-length sequences of influenza A segments indicated that sequences from the National Institutes of Health Influenza Genome Sequencing Project (IGSP), which were generated under stricter quality control procedures than other published influenza virus sequences, included only two phylogenetically supported single recombinant sequences and no recombinant clades that would be indicative of circulating recombinant forms. The non-IGSP data, on the other hand, showed a much larger amount of potential recombination [12]. Because sample handling and sequencing are executed to a very high standard in the IGSP these sequences should be less likely to be exposed to contamination by other samples or laboratory strains, and thus should be less likely to exhibit laboratory-generated signals of homologous recombination. Conversely, the higher frequency of putative recombinants in less stringently generated influenza virus sequences is likely due to artifacts.

Additional unpublished results have confirmed that influenza virus recombinant sequences are often generated artificially. The NP gene sequences of A/chicken/Shantou/5738/2001(H5N1) (GenBank accession No. CY029091) and A/chicken/Shantou/28/2002(H5N1) (GenBank accession No. CY029112) initially showed strong homologous recombination signals. However, when the two NP genes were re-sequenced from original virus stocks, each new sequence differed in the first 500–600 bp from the ones in GenBank [72] and the apparent recombination was revealed to be due to laboratory contamination and/or sequencing errors (both published sequences have now been replaced by the corrected ones). Hence, sequences in public repositories, though valuable for exploratory purposes, are not generally sufficient for producing definitive conclusions of recombination in influenza viruses.

Given the evidence to date, homologous recombination seems to play little or no role in the evolution of influenza viruses in natural settings.

### 4.2. Ebola Virus

One potentially interesting example of recombination in NSVs is a lineage of Zaire ebolavirus reported to have a hybrid origin [48]. Zaire ebolavirus is one of the most virulent human pathogens, causing acute hemorrhagic fever that can lead to death in a matter of days, with a very high fatality rate. The putative recombination event was inferred to have occurred between 1996 and 2001 giving rise to a group of recombinant viruses that were responsible for a series of outbreaks in 2001–2003 [48]. However, this evidence was only based on phylogenetic analyses of two distinct genes, glycoprotein (GP) and NP. The detection of at least nine genetically distinct lineages of Marburg virus that belong to the genus of ebolavirus in circulation during the outbreak of Marburg hemorrhagic fever most intensively in 1999 suggests that co-infection with multiple filovirus strains is a possibility [73]. If samples contain multiple virus strains, PCR amplification of distinct genomic regions can lead to different strains being sequenced for different genomic regions and the generation of a group of artificial recombinant sequences is possible. Therefore, additional experiments would be necessary to confirm this recombination event, preferably by pinpointing the actual recombination breakpoint(s) within a single amplicon.

### 4.3. Newcastle Disease Virus

Between the 1950s and 1960s, extensive co-infection experiments with NDV using a variety of markers failed to reveal any evidence of recombination [30]. These experiments led to the conclusion that recombination might not be a feature of the biology of NDV. With the advent of modern molecular biology techniques, however, many NDV gene or genome sequences have become available and many recombination events have been reported [33,61–67]. Laboratory contamination is of particular concern here because vaccine derived strains contributed the majority of the recombinant regions, and these strains might well have been present in laboratories sequencing field strains of NDV. Chong *et al*. argued that the presence of unique nucleotide substitutions in the recombinant regions compared to the comparable region of the predicted parental genotypes suggests that these regions did not arise due to contamination with vaccine strains deposited in the sequence databases [65]. However, the widespread use of live vaccines in poultry and the extensive presence of non-virulent endemic NDVs in live bird markets and in wild animals make the existence of unnoticed mixed infections in field samples a strong possibility [74].

With frequent mixed infections of NDV (in 5–15% of wildlife isolates and perhaps much higher levels in vaccinated poultry) there is a high likelihood of having two or more viruses in the same inoculum during egg-amplification during initial isolation. Although plaque purification steps were performed in some of these studies, no attempts were made to confirm the absence of contaminating viruses in the original samples or to investigate the possibility of contamination with PCR products, which normally abound in NDV sequencing laboratories [75]. Some of the apparent recombinants available in GenBank have now been confirmed to be artificially generated ones [76,77].

However, several viruses isolated from 1971 through 2006 in North America share a virtually identical breakpoint. Because most of these viruses were isolated independently at different time points and/or locations, this event appears to have occurred naturally and not as the result of a laboratory artifact [67]. The details of these recombinants should be further investigated in the future. More importantly, whole genome sequencing following co-infection experiments, possibly between wild-type and vaccine strains, would be useful for determining experimentally whether recombination can indeed occur in NDV and how frequent it may be.

### 4.4. Hantavirus

To date, the strongest evidence for recombination in NSVs comes from hantaviruses. Hantaviruses constitute a genus in the *Bunyaviridae* family. Phylogenetic analysis of the S segment indicated that the eastern Slovakian Tula hantavirus underwent homologous recombination and formed an independent lineage [41]. A later transfection-mediated rescue of Tula virus generated a recombinant S RNA segment and independent attempts yielded S RNA molecules of similar structure with a breakpoint located close to one of the breakpoints suggested for the natural recombinants [31]. The possibility of the occurrence of such similar breakpoints seems exceedingly low. Thereafter, several reports of recombination in members of the hantavirus genus followed, including Hantaan virus, Dobrava virus, Andes virus, and Puumala virus [42–47]. Therefore, recombination appears to be a common occurrence in hantaviruses, relative to most other NSVs. This suggests there are unknown mechanisms contributing to hantavirus recombination that might be interesting in their own right and also potentially informative about why other NSVs do not appear to undergo much recombination.

### 4.5. Arenavirus

Phylogenetic analyses based on the viral glycoprotein precursor (GPC) and the nucleoprotein (NP) encoded by the S RNA segment suggest a common hybrid origin of Catarina virus, Skinner Tank virus, Whitewater arroyo virus, Tamiami virus, and Bear canyon virus, defined as the A/Rec lineage [34–38]. However, an alternative explanation based on the peculiarity of the GP1 region within the GPC gene has been proposed [37]. GP1 is highly variable in clades B and the A/Rec lineage and the problematic alignment of the GP1 regions represents a potentially confounding factor for these phylogenetic analyses [37]. Nevertheless, when only the most conserved regions of GPC are used, the inconsistency of relationships between North American arenaviruses and clades A and B arenaviruses is still present [37,38]. We believe is it plausible that, like the spurious signal of recombination in the 1918 Spanish Influenza hemagglutinin gene, the phylogenetic information derived from the arenavirus GPC is influenced by specific selection pressures [36]. Given the phenomenon of superinfection exclusion and the absence of any lab-created or characterized arenavirus recombinants, the possibility of recombination in arenaviruses remains unsubstantiated [37,78–80]. Therefore, more studies are needed to attempt to distinguish phylogenetic signals generated by recombination and selection.

### 4.6. Summary

Most of the reported cases of recombination in NSVs appear to be artifacts upon closer scrutiny. While recombination clearly does occur in some NSVs, such as hantaviruses and RSV, homologous recombination is evidently generally rare in NSVs.

## 5. Constraints on Recombination in Negative Sense RNA Viruses

Why is the recombination rate so low in negative sense RNA viruses? While it is difficult to provide a definitive answer to this crucial question, several factors may contribute to it. The viral RNA of NSVs is condensed by the nucleoprotein into a helical nucleocapsid, which is called ribonucleoprotein complex (RNP) and which serves as a suitable template for replication. Unlike cores, capsids, or nucleocapsids of positive-strand RNA viruses or DNA viruses, RNPs apparently never disassemble, and RNA synthesis does not change the structure of the RNA-N protein template [81–84]. The RNP may thus be a barrier to RNA recombination. On the other hand, the production of defective interfering (DI) RNAs indicates that the polymerase is able to jump off and on to the template of RNA molecules relatively frequently [29].

Recombination is generated by the exchange of genetic material between viruses that co-infect individual host cells. Therefore, a low rate of co-infection will result in a low rate of recombination. Several factors could limit virus co-infection. For example, negative-sense RNA viruses tend to produce acute infections with relatively short recovery periods. While recombination in hantaviruses seems to be the best evidence yet observed, it is worth noting that hantaviruses can establish persistent infections in their rodent hosts [41,46]. Superinfection exclusion, in which a primary viral infection prevents a secondary infection with the same or closely related virus, is another potentially recombination-limiting feature that has been observed in several negative sense viruses, such as Borna disease virus, measles virus, and vesicular stomatitis virus [85,86].

Another possible reason for a low level of observed recombinants is the potentially low fitness of recombinants, which will be selected against. The recombinant Tula hantavirus has been observed to be less competitive than the wild type [31,87], though why NSV recombinants should be less fit generally than recombinants in other viral lineages is not clear.

## 6. Implications

Viral sequences have been widely used for phylogenetic analyses to decipher the evolutionary dynamics, determine the genotypes, and trace the spread of viruses. However, both natural and artificial recombination are routinely ignored in most of these phylogenetic analyses. Ignoring recombination might severely compromise phylogenetic analyses, because different genomic regions will have different evolutionary histories [88]. We suggest that recombination detection should be a standard component of every phylogenetic analysis of negative sense RNA viruses. Even though the evidence to date suggests recombination may be rare, such analyses also serve an important quality-control function by helping to weed out laboratory and analytical errors.

There are also practical implications of recombination in NSVs. For instance, NDV has recently been proposed as a potential vector in the development of a novel bi- or multivalent vaccine [89–91]. The sporadic reports on recombination in non-segmented negative sense RNA viruses have led to cautions about safety issues with respect to the use of NDV-based live attenuated vaccines [92]. This issue is under intense debate at the moment [75,92–94]. Although the evidence for recombination is not overwhelming to date [75], recombination cannot be excluded, especially due to the discovery of a lineage with virtually identical breakpoint [67]. Recombination between a vaccine virus and other circulating non-segmented negative-strand RNA viruses resulting in unexpected recombinants and the potential instability of the inserted foreign gene should be fully addressed and evaluated.

## 7. Conclusions and Further Directions

Sporadic authentic examples suggest that homologous recombination does occur in NSVs. However, homologous recombination seems to be very rare or even absent in most NSVs. Most of the recombination events reported in the literature appear to have been generated artificially due to laboratory contamination or unsuitable bioinformatics methods. Therefore, putative homologous recombination events in NSVs should be treated with caution. Providing detailed explanations of why possible cases should be believed to be authentic recombination should be standard practice when reporting recombination in NSVs. Similarly, co-infection experiments should be performed to determine experimentally whether homologous recombination occurs in NSVs and at what rate NSVs recombine, if at all. If no recombination is observed in the most permissive laboratory settings with a particular virus, this is all the more reason to treat apparent cases of natural recombination in wild viruses of the same type with suitable skepticism.

## Figures and Tables

**Table 1.** List of reported recombination in negative sense RNA viruses.

**Virus**	**Type**	**Family**	**Evidence**	**Reference**
Arenavirus	Segmented	Arenaviridae	Sequence	[34–38]
Crimean-Congo hemorrhagic fever virus	Segmented	Bunyaviridae	Sequence	[39,40]
Hantaviruses	Segmented	Bunyaviridae	Sequence/Transfection	[31,41–47]
Zaire ebolavirus	Nonsegmented	Filoviridae	Sequence	[48]
Infectious salmon anemia virus	Segmented	Orthomyxoviridae	Sequence	[49,50]
Influenza A virus	Segmented	Orthomyxoviridae	Sequence/Coinfection	[20,51–55]
Canine distemper virus	Nonsegmented	Paramyxoviridae	Sequence	[56,57]
Respiratory syncytial virus	Nonsegmented	Paramyxoviridae	Sequence/Coinfection	[32,58]
Measles virus	Nonsegmented	Paramyxoviridae	Sequence	[59]
Mumps virus	Nonsegmented	Paramyxoviridae	Sequence	[13,33]
Human parainfluenza virus	Nonsegmented	Paramyxoviridae	Sequence	[60]
Newcastle disease virus	Nonsegmented	Paramyxoviridae	Sequence	[33,61–67]
Rabies virus	Nonsegmented	Rhabdoviridae	Sequence	[68]
